# Full-term pregnancy after severe gestational psittacosis: a case report and literature review

**DOI:** 10.3389/fphar.2026.1836961

**Published:** 2026-06-23

**Authors:** Zhe Zhao, Ming Lu, Yingqiu Ying

**Affiliations:** 1 Department of Pharmacy, Peking University Third Hospital, Beijing, China; 2 Institute for Drug Evaluation, Peking University Health Science Center, Beijing, China; 3 Department of Respiratory and Critical Care Medicine, Peking University Third Hospital, Beijing, China; 4 Infectious Disease Center, Peking University Third Hospital, Beijing, China

**Keywords:** *Chlamydia psittaci*, gestational psittacosis, metagenomic next-generation sequencing, multidisciplinary care, term pregnancy

## Abstract

Gestational psittacosis is a rare but high-risk infection caused by *Chlamydia psittaci*, often leading to severe maternal complications and adverse fetal outcomes. We report a unique case of a 30-year-old woman at 22 + 5 weeks of gestation who presented with acute high fever and respiratory failure following bird exposure. The diagnosis of *C. psittaci* infection was rapidly confirmed via blood metagenomic next-generation sequencing (mNGS). Following multidisciplinary consultation involving obstetricians, infectious disease specialists, intensivists, respiratory physicians, clinical pharmacists, and neonatologists, an individualized management plan was established to balance maternal infection control, respiratory support, fetal monitoring, and medication safety during pregnancy. The patient was treated with intravenous azithromycin combined with corticosteroids, and her clinical condition stabilized within 2 weeks. Notably, the pregnancy continued to term, resulting in the delivery of a healthy male infant. To our knowledge, this represents the first reported case worldwide of a successful full-term delivery following gestational psittacosis. This case underscores the critical importance of early mNGS-based diagnosis, multidisciplinary collaboration, and appropriate antimicrobial therapy in optimizing maternal and neonatal outcomes, providing a valuable clinical reference for managing this life-threatening zoonosis during pregnancy.

## Introduction

Psittacosis is a zoonotic infection caused by *Chlamydia psittaci* (*C. psittaci*), typically acquired through inhalation of aerosolized secretions or feces from infected birds (Huang et al., 2023; Wang et al., 2023). Although considered uncommon, psittacosis accounts for approximately 1%–2% of community-acquired pneumonia cases. Its clinical spectrum is broad, ranging from mild influenza-like illness to fulminant severe pneumonia complicated by sepsis and multi-organ dysfunction (Yang et al., 2022). Severe disease is well recognized, with reported intensive care unit (ICU) admission rates of up to 20%–30% and case-fatality rates ranging from 1% to 5%, the latter increasing significantly in cases complicated by respiratory failure or sepsis (Yang et al., 2021; Wang et al., 2023). Delayed recognition or initiation of effective anti-chlamydial therapy are major contributors to disease progression. Severe outcomes are more likely to occur in patients with advanced age, underlying comorbidities, immunocompromised status, high pathogen burden, or progression to severe pneumonia, respiratory failure, sepsis, disseminated intravascular coagulation, and multi-organ dysfunction (Yang et al., 2021; Wang et al., 2023).

Pregnancy represents a high-risk state for psittacosis. Gestational psittacosis is reported as a rare but extremely severe disease, with significant maternal mortality (10%–15%) and fetal mortality exceeding 70%–80% (Katsura et al., 2020). Maternal death is usually associated with rapidly progressive severe pneumonia, respiratory failure or acute respiratory distress syndrome, sepsis-related complications, disseminated intravascular coagulation, shock, and multi-organ dysfunction. These risks may result from pregnancy-associated immunological and cardiopulmonary adaptations, which reduce maternal tolerance to severe pneumonia, hypoxemia, and sepsis (Lopes van Balen et al., 2017; Hegewald and Crapo, 2011). Besides, fetal death may be caused by placental involvement of *C. psittaci*, which lead to placentitis, impaired uteroplacental perfusion, fetal hypoxia, intrauterine infection, or secondary effects of maternal critical illness (Nishino et al., 2026). Treatment is particularly challenging because tetracyclines, the standard first-line therapy for psittacosis in nonpregnant adults, are generally contraindicated during pregnancy due to potential fetal bone and dental toxicity. Therefore, macrolides, such as erythromycin or azithromycin, are considered pregnancy-compatible alternatives before delivery despite limited available evidence (Katsura et al., 2020). Furthermore, due to its non-specific presentation and the limitation of conventional diagnostic approaches, gestational psittacosis is frequently underdiagnosed or diagnosed late, especially when routine microbiological tests are negative. Recently, metagenomic next-generation sequencing (mNGS) has emerged as a valuable diagnostic tool for identifying etiology, enabling accurate and early diagnosis of psittacosis. However, reports of mNGS-guided diagnosis and successful treatment of gestational psittacosis with favorable fetal outcomes remain scarce.

Here, we report a severe case of gestational psittacosis that was promptly diagnosed by blood mNGS and resulted in full-term delivery. We also summarize the key clinical features from published gestational psittacosis cases through a literature review.

## Case presentation

A 30-year-old pregnant woman at 22+5 weeks of gestation presented with 1-week history of urinary frequency and urgency, a high fever for 4 days, and acute-onset respiratory distress for 10 h before admission. One week prior to admission, the patient developed urinary frequency, urgency, and dysuria but did not seek medical attention. Four days before admission, she experienced sudden onset of high fever up to 39.4 °C. Initial laboratory tests revealed a white blood cell (WBC) count of 8.74 × 10^9^/L, neutrophil percentage of 84.2%, platelet count of 138 × 10^9^/L, and C-reactive protein (CRP) level of 23.59 mg/L. According to the available pre-admission medical records, no antimicrobial treatment was initiated at that visit. Three days later, her fever escalated to 40.4 °C, with urinalysis showing leukocyte positivity. Repeat blood tests revealed WBC 6.62 × 10^9^/L, neutrophil percentage 84.6%, platelet count102 × 10^9^/L, and CRP 37.4 mg/L. Tests for SARS-CoV-2, influenza A and B were all negative. Based on the urinary symptoms and urinalysis findings, a presumptive diagnosis of urinary tract infection was made, and she was treated with acetaminophen and oral cefdinir. Subsequent evaluation at an outside hospital demonstrated progressive thrombocytopenia (platelets decreased to 63 × 10^9^/L), elevated CRP (37.4 mg/L), procalcitonin (1.81 ng/mL), and brain natriuretic peptide (BNP) (638.2 pg/mL). She received intravenous cefoperazone–sulbactam and urinary catheterization. Approximately 10 h prior to admission, the patient developed sudden respiratory distress, accompanied by cough, sputum production, chest tightness, and recurrent fever up to 39 °C, prompting urgent transfer to our hospital. Obstetric history of the patient was gravida 3, para 1 with one prior full-term infant vaginal delivery of a healthy infant and two induced abortions. Her menstrual cycles were regular, and fetal movements had been perceived and active for 18 weeks of gestation. The pregnancy had been uncomplicated until the onset of this illness. Her past medical and family histories were unremarkable. On physical examination, the patient appeared acutely ill, with a distressed facial expression and orthopneic posture. She was alert and oriented. The abdomen was soft, non-tender, and without costovertebral angle tenderness. Lung auscultation revealed bilateral crackles without wheezes. However, she initially declined chest radiography due to concerns about potential radiation exposure to the fetus.

**FIGURE 1:**
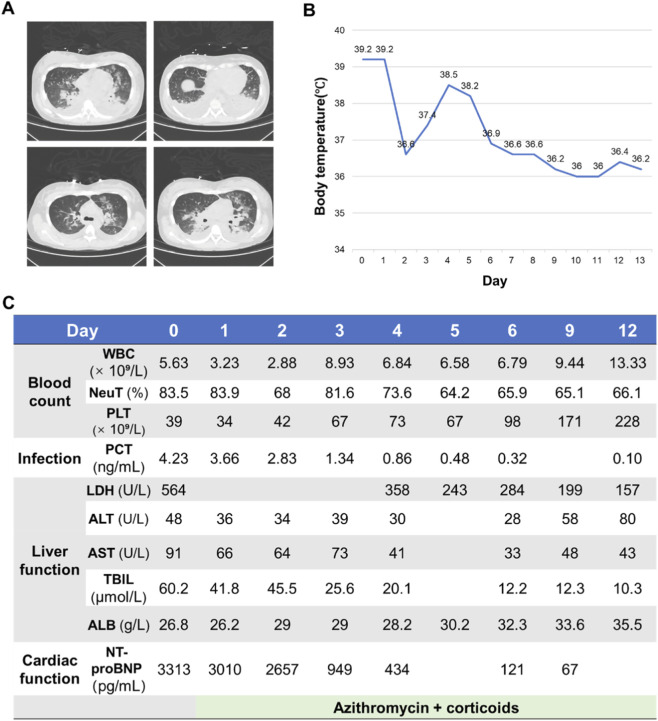
Clinical course and dynamic changes in radiological and laboratory findings. **(A)** Chest computed tomography images at admission. **(B)** Temporal changes in inflammatory markers during hospitalization. **(C)** Trends in laboratory results following initiation of azithromycin and corticosteroid therapy. WBC, white blood cell count; NeuT%, neutrophil percentage; PLT, platelet count; PCT, procalcitonin; LDH, lactate dehydrogenase; ALT, alanine aminotransferase; AST, aspartate aminotransferase; TBIL, total bilirubin; ALB, albumin; NT-proBNP, N-terminal pro–brain natriuretic peptide.

After admission to our hospital, the patient was reassessed based on the full disease course and physical examination. Given the persistent high fever, acute respiratory symptoms, and poor general condition, the initial clinical impression was severe infection with sepsis, with pulmonary infection or atypical pneumonia suspected. Besides, the initial differential diagnoses included severe community-acquired pneumonia, atypical pathogen infection, and viral pneumonia. On admission, laboratory investigations showed a WBC count of 5.63 × 10^9^/L, neutrophil percentage of 83.5%, and severe thrombocytopenia (platelet count of 39 × 10^9^/L). Biochemical tests revealed markedly elevated procalcitonin (4.23 ng/mL) and N-terminal pro–brain natriuretic peptide (NT-proBNP, 3,313 pg/mL), along with liver dysfunction (ALT 48 U/L, AST 91 U/L, total bilirubin 60.2 μmol/L), elevated lactate dehydrogenase (564 U/L), and hypoalbuminemia (26.8 g/L). Blood, urine and sputum cultures showed no bacterial growth. Given the patient’s severe symptoms, blood mNGS was performed immediately after admission. The result became available within 24 h and identified *C. psittaci*, with 93,468 specific sequencing reads detected. Upon further questioning, the patient reported that she had fed seagulls during a trip to Jeju Island, South Korea, 3 weeks prior. She also recalled visiting a hair salon 2 weeks before admission where several parrots were kept; notably, two of the parrots had died shortly before her visit. This finding suggested possible active infection among the birds and environmental contamination in an enclosed setting, although no microbiological testing of the birds or environment was performed. After *C. psittaci* was detected by blood mNGS and severe pulmonary infection was suspected, the necessity of chest CT to assess the severity and extent of pneumonia and guide urgent management was explained to the patient and her family. With appropriate radiation protection measures and continued obstetric monitoring, chest CT was performed and showed bilateral multifocal pulmonary infiltrates, ground-glass opacities, and consolidation, consistent with severe pneumonia (Figure 1A).

Based on the clinical presentation, imaging findings, bird exposure history, pregnancy status, and positive mNGS results, the patient was diagnosed with gestational psittacosis complicated by severe pneumonia and acute cardiac insufficiency. A multidisciplinary team involving infectious disease specialists, clinical pharmacists, and obstetricians was promptly convened to guide management. She was treated with intravenous azithromycin (500 mg daily) as pregnancy-compatible targeted therapy, in combination with systemic corticosteroids (dexamethasone, at a dose of 10 mg on the first day, followed by 5 mg daily for the next 4 days). Supportive care included non-invasive positive pressure ventilation, anticoagulation, mucolytics, and gastrointestinal protection. After 2 weeks of treatment, the patient became afebrile, with marked improvement in respiratory symptoms, and gradual resolution of laboratory abnormalities (Figures 1B-C). Follow-up assessments indicated an uncomplicated ongoing pregnancy, and she later delivered a healthy full-term male infant vaginally. At the latest available follow-up, the infant remained well, with no respiratory symptoms, developmental concerns, or infection-related complications reported beyond the neonatal period; longer-term monitoring of growth and neurodevelopment is ongoing.

## Patient perspective

The patient reported significant anxiety during the early stage of illness because of persistent high fever, respiratory distress, and concern about fetal safety. After the diagnosis was confirmed and a multidisciplinary treatment plan was established, she felt reassured by the close monitoring of both maternal and fetal conditions. Following recovery and the subsequent full-term vaginal delivery of a healthy infant, she expressed gratitude for the timely diagnosis and coordinated care, and agreed that sharing her experience may help raise awareness of gestational psittacosis.

## Literature review

**FIGURE 2:**
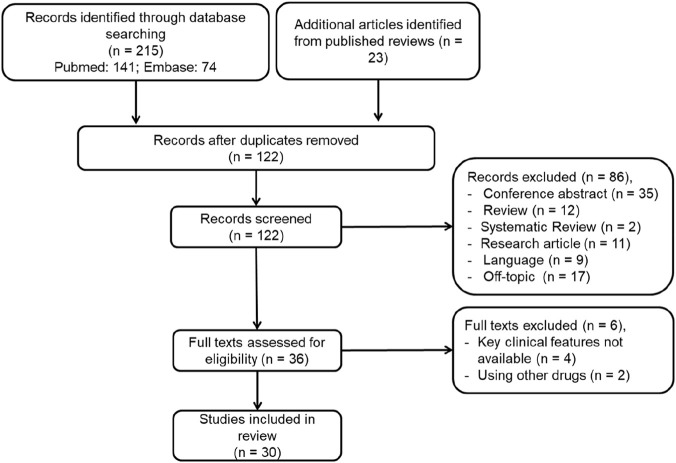
Flow chart of study selection for the literature review of gestational psittacosis.

To better understand this severe infection during pregnancy, we conducted a comprehensive literature review of gestational psittacosis cases by searching the PubMed and Embase databases using combination of keywords related to pregnancy and psittacosis. After screening and evaluation (Figure 2), a total of 30 eligible cases were included for analysis (Table 1) (Wang et al., 2023; Miyauchi et al., 2024; Yoshimura et al., 2022; Guscoth et al., 2022; Corsaro and Greub, 2006; Sun et al., 2021; Burgener et al., 2022; Katsura et al., 2020) and a detailed summary of their clinical features is shown in Table 1. In this review, the pregnant patients ranged in age from 20 to 41 years old. Their gestational ages at diagnosis varied from 6 to 36 weeks, with a mean of 24.7 weeks. Notably, one patient was diagnosed in the first trimester, while 16 cases were identified in the second trimester and 13 in the third trimester. Among these cases, common clinical manifestations included fever (29/30), headache (18/30), and cough (12/30). Frequently observed complications comprised thrombocytopenia (26/30), hepatic dysfunction (19/30), and renal dysfunction (17/30). Among the 24 patients who received antibiotic therapy, macrolides (erythromycin or clarithromycin) were administered to 41.7% (10/24), while tetracyclines (tetracycline or doxycycline) were utilized in 58.3% (14/24) of cases. The overall mortality rate among 30 patients was 13.3% (4/30), and the fetal mortality rate of 80.0% (24/30). All six surviving fetuses (6/30) were delivered preterm. A focused comparison between fetal survival and fetal death cases was further conducted. The mean gestational age at diagnosis was significantly higher in cases with fetal survival (31.3 ± 0.7 weeks) compared to those with fetal demise (23.0 ± 1.4 weeks; P = 0.008), demonstrating a critical association between later gestational age at infection and improved fetal outcomes. Nevertheless, antibiotic class did not appear to fully explain fetal outcome, as both macrolides and tetracyclines were used in cases with fetal survival and fetal death.

**TABLE 1:** Clinical characteristics of gestational psittacosis cases.

Patients	1	2	3	4	5	6	7	8	9	10	11	12	13	14	15	16	17	18	19	20	21	22	23	24	25	26	27	28	29	30
Age (years)	31	36	20	25	28	32	25	30	35	32	​	31	26	26	29	24	28	22	30	29	31	34	34	41	28	26	​	33	27	33
Source	P	S	S	S	S	G	S	S	S	S	G	P	P	G	S	S	S	S	S	S	S	S	S	Pigeon	—	—	S	P	Poultry	S
Gestational age (weeks) at first visit	17	31	26	19	28	16	22	15	19	6	32	31	32	32	27	14	28	24	27	28	25	34	36	16	28	23	31	29	26	19
Symptoms																														
Fever	+	+	+	+	+	+	+	+	+	+	+	+	+	+	+	+	+	​	+	+	+	+	+	+	+	+	+	+	+	+
Headache	+	+	+	+	+	​	+	​	​	​	​	+	+	+	+	+	+	​	​	+	+	+	+	​	​	​	+	+	​	​
Nausea	​	​	+	​	+	​	+	​	+	​	​	+	+	​	​	​	+	+	​	​	+	​	+	​	​	+	​	​	​	​
Cough	​	​	​	​	​	​	+	+	+	+	​	+	+	+	​	+	​	​	​	​	​	​	​	+	​	​	​	+	+	+
Sore throat	​	​	​	​	+	​	​	​	​	​	​	​	+	​	+	​	​	​	​	​	+	​	​	​	​	​	​	​	​	​
Chest pain	​	​	​	​	​	​	​	+	​	+	​	+	​	​	​	​	​	+	​	+	​	+	​	​	​	​	​	​	​	​
Abdominal pain	​	+	+	+	​	​	​	​	+	​	​	​	+	​	​	+	​	​	​	​	​	+	+	​	​	​	​	​	​	​
Back pain	​	​	​	+	​	​	​	​	​	​	​	+	​	​	​	+	​	+	​	​	​	​	+	​	​	​	​	​	​	​
Complications																														
Thrombocytopenia	+	+	+	+	+	+	+	+	+	+	+	−	+	+	+	+	​	​	+	+	+	+	+	+	+	+	+	​	+	+
Hepatic dysfunction	+	+	+	+	+	​	+	+	+	​	+	​	+	+	+	​	+	−	​	​	+	​	​	+	​	+	+	​	+	+
Renal dysfunction	+	+	​	+	+	​	+	​	−	​	+	​	−	+	+	​	+	+	+	​	+	​	​	​	​	​	+	​	+	+
DIC	+	​	​	​	+	​	+	+	​	+	​	​	+	​	+	​	​	​	​	​	+	+	+	​	​	​	+	​	​	​
Chest X-ray/CT findings	​	−	​	−	​	​	+	+	−	+	+	+	+	​	​	​	​	−	​	​	​	−	​	CT+	CT+	−	CT+	CT+	+	​
Subsequent progress																														
EM/CAM	−	−	​	​	−	+	​	+	−	+	​	+	+	−	+	+	−	−	​	−	+	+	−	−	−	​	+	−	−	−
TC/DOXY	−	+	​	​	−	+	​	+	+	+	​	+	−	−	+	−	−	+	​	+	+	−	−	−	−	​	+	+	+	+
Fetal death	+	−	+	+	+	+	+	+	+	+	+	−	−	+	+	+	+	+	+	+	+	−	+	+	+	+	−	−	+	+
Maternal death	+	−	−	−	−	−	−	−	−	−	−	−	−	−	−	−	−	−	−	−	−	−	+	−	+	+	−	−	−	−
Placental infection	​	+	+	+	+	​	​	+	+	​	​	​	+	+	+	+	+	+	​	​	+	​	​	​	+	+	​	−	​	+
Fetal infection	​	−	−	​	+	​	​	−	​	​	​	​	+	−	−	+	+	​	​	​	​	​	​	​	​	​	​	​	​	​

Fever was defined as body temperature ≥38.0 °C. Thrombocytopenia, hepatic dysfunction, and renal dysfunction were defined according to reported abnormal laboratory findings or the original authors’ descriptions. + = present, − = absent, blank = not described. CAM, clarithromycin; DIC, disseminated intravascular coagulation; DOXY, doxycycline; EM, erythromycin; G, goat; P, parrot; S, sheep; TC, tetracycline.

## Discussion

Gestational psittacosis is a rare but life-threatening zoonotic infection caused by *C. psittaci* (Huang et al., 2023; Wang et al., 2023). We report a severe case diagnosed by blood mNGS complicated by respiratory failure, and multi-organ involvement. Timely pregnancy-compatible antimicrobial therapy combined with multidisciplinary care resulted in complete maternal recovery and full-term delivery of a healthy infant. A literature review of published gestational psittacosis cases is also presented.

Following infection of *C*. *psittaci*, the incubation period typically ranges from 5 to 14 days (Zhang et al., 2022). The disease most commonly presents with high fever, chills, headache, unproductive cough, and may manifest as influenza-like illness or atypical pneumonia (Yang et al., 2022). A diagnosis of psittacosis can be established in patients with a clear history of avian exposure, compatible clinical features, abnormal pulmonary imaging, and microbiological confirmation. Although the disease course is relatively mild in most non-pregnant individuals, some patients experience rapid deterioration with systemic involvement (Yang et al., 2021; Wang et al., 2023). Psittacosis during pregnancy is particularly severe and may result in life-threatening complications, including respiratory failure, hepatic dysfunction, sepsis, and disseminated intravascular coagulation. Moreover, *C. psittaci* is capable of crossing the placental barrier and proliferating within placental tissue, leading to impaired uteroplacental perfusion, fetal distress, miscarriage, or intrauterine fetal demise (Katsura et al., 2020). Published literature has consistently demonstrated devastating prognosis associated with gestational psittacosis. Reported fetal mortality rates have reached as high as 70-80%, while maternal mortality ranges from approximately 10-15% (Katsura et al., 2020).

In our updated review of 30 cases, thrombocytopenia, hepatic dysfunction, and renal dysfunction were observed in 26/30 (86.7%), 19/30 (63.3%), and 17/30 (56.7%) cases, respectively, while fetal death and maternal death occurred in 24/30 (80.0%) and 4/30 (13.3%) cases. Potential risk factors for complications in gestational psittacosis may include recent exposure to birds or livestock, delayed diagnosis or delayed initiation of effective anti-chlamydial therapy, persistent high fever, influenza-like symptoms, normal or only moderately abnormal leukocyte count, placental involvement, and earlier gestational age at infection. Moreover, our comparison of fetal survival and fetal death cases further indicated that later gestational age at diagnosis was strongly associated with fetal survival. This association may have several explanations. First, a more mature fetus may better tolerate transient maternal systemic inflammation, hypoxemia, or hemodynamic instability. Second, when infection occurs in late pregnancy, emergency delivery can be considered if maternal or fetal status deteriorates, whereas this option is limited at earlier gestational ages. Third, earlier gestational infection may allow more time for placental colonization, placentitis, and impaired uteroplacental perfusion before effective therapy is initiated. Previous reports have suggested that fetal death can occur even without confirmed fetal infection, indicating that placental infection and uteroplacental perfusion failure may be critical mechanisms of fetal demise. However, direct evidence that placental permeability or transplacental transmission of *C. psittaci* differs according to gestational age remains limited. Future studies incorporating placental pathology, placental and fetal microbiological testing, and neonatal follow-up are needed to clarify this issue.

The present case aligns well with previously reported clinical features of gestational psittacosis, including high fever, headache, cough, thrombocytopenia, and hepatic and renal dysfunction. Notably, the patient initially presented with urinary tract infection-like symptoms, including urinary frequency, urgency, and dysuria, with leukocyte positivity on urinalysis. Because urine culture was not obtained before antibiotic exposure, a concomitant urinary tract infection could not be completely excluded. However, after admission, urine culture showed no bacterial growth, whereas blood mNGS identified *C. psittaci*, and the subsequent clinical course was dominated by severe pneumonia, respiratory failure, and systemic infection. Therefore, gestational psittacosis was considered the primary diagnosis underlying the severe systemic illness. Furthermore, despite infection occurring during the second trimester, a period traditionally associated with particularly poor fetal prognosis, the patient achieved a favorable outcome. This successful outcome can be attributed to early etiological diagnosis via mNGS, which enabled timely pathogen-directed therapy, as well as close multidisciplinary collaboration. *Chlamydia psittaci* is an obligate intracellular pathogen that is difficult to identify through conventional *in vitro* methods in routine laboratory settings. Commercially serological assays and standardized PCR diagnostic kits remain unavailable in many countries, including China. In the presenting case, the patient presented with persistent high fever without an identifiable source of infection. Empirical therapy with cephalosporin proved ineffective, and concurrent blood and urine cultures yielded no growth. Given the severity of illness and the absence of a definitive etiological diagnosis, mNGS of blood was performed, which ultimately identified *C. psittaci,* enabling timely initiation of pathogen-directed therapy. Although chest imaging was initially deferred due to concerns regarding fetal radiation exposure, a chest CT was subsequently performed once psittacosis was strongly suspected, which confirmed extensive pulmonary involvement. To our knowledge, this is the first reported case of confirmed gestational psittacosis resulting in full-term delivery of a healthy infant, representing a notable deviation from previously reported outcomes.

Appropriate antimicrobial selection during pregnancy represents a major challenge in the management of gestational psittacosis. In non-pregnant patients, tetracyclines are considered first-line therapy (Pariente et al., 2016). However, they are classified as pregnancy category D by the U.S. Food and Drug Administration and are associated with significant maternal hepatotoxicity as well as adverse fetal effects, including impaired bone growth and tooth discoloration (Ghanshani et al., 2025; Nakitanda et al., 2024). In our review, non-macrolide therapy, like tetracycline or doxycycline, were selected in some pregnant patients with psittacosis. Their use in previous reports may be explained by the fact that tetracyclines are the most effective first-line agents for psittacosis in nonpregnant adults, the diagnosis was sometimes made only after fetal demise or delivery, and maternal disease was often life-threatening. In addition, some cases were reported before current pregnancy-compatible treatment preferences were widely adopted. Therefore, treatment heterogeneity across previous cases likely reflected differences in diagnostic timing, fetal status, disease severity, and historical practice patterns. Consequently, tetracyclines are generally contraindicated during pregnancy. Fluoroquinolones are also controversial in this population due to concerns regarding fetal cartilage and skeletal toxicity demonstrated in animal studies (Aboubakr et al., 2014; Shakibaei et al., 2002). Current expert consensus and clinical guidelines therefore recommend macrolide antibiotics as the preferred treatment for psittacosis during pregnancy. In our literature review, macrolides were used in approximately 42% of treated cases. Although fetal outcomes remained poor overall, maternal survival was preserved in all macrolide-treated patients. In the present case, azithromycin was selected as a pregnancy-compatible agent and proved effective in controlling infection, supporting its role as a first-line option in the management of gestational psittacosis.

In addition to targeted antimicrobial therapy, systemic corticosteroids played a critical role in the management of this patient. Severe community acquired pneumonia is characterized by an excessive inflammatory response that can lead to extensive lung injury, acute respiratory distress syndrome (ARDS), and increased mortality. Clinical guidelines for severe community-acquired pneumonia recommend early corticosteroid therapy in patients with respiratory failure requiring ventilatory support or those with significant hypoxemia (Metlay et al., 2019). Nevertheless, current evidence for corticosteroid use in gestational psittacosis remains limited, and no data indicate that the indication of corticosteroid therapy should vary according to gestational age. In practice, corticosteroid decisions during pregnancy are generally individualized according to maternal disease severity, respiratory status, hypoxemia, systemic inflammatory burden, hemodynamic instability, risk of preterm delivery, and fetal safety considerations. In this case, corticosteroids were administered because of severe pneumonia, respiratory distress, and marked systemic inflammation after multidisciplinary risk–benefit assessment. Further studies are needed to define the optimal role, timing, dose, and safety of corticosteroids in gestational psittacosis.

Overall, this case demonstrates that early recognition of gestational psittacosis, prompt application of sensitive diagnostic tools such as metagenomic next-generation sequencing (mNGS), and individualized treatment guided by multidisciplinary collaboration are critical to improving maternal and fetal outcomes. The successful full-term delivery achieved in this case underscores the importance of pregnancy-compatible antimicrobial therapy and judicious use of corticosteroids in the management of severe infection during pregnancy. By systematically summarizing previously reported cases and presenting this rare favorable outcome, our report enhances current understanding of gestational psittacosis and provides valuable evidence to support optimized diagnostic and therapeutic strategies in this high-risk population. Nevertheless, several limitations should be acknowledged. Gestational psittacosis is extremely rare, and current evidence is mainly based on case reports and small case series, which may be affected by publication bias and incomplete reporting. The included cases also span several decades, during which diagnostic methods, pathogen nomenclature, imaging practices, and treatment strategies have changed, limiting comparability across reports. In addition, some cases lacked key clinical information, such as exposure history, placental or fetal infection, antimicrobial timing, and neonatal follow-up, limiting more detailed pooled analyses. Finally, as the present case report describes a single patient, the favorable outcome cannot be attributed to any single intervention.

Future studies should establish multicenter registries for gestational psittacosis, adopt standardized diagnostic and reporting criteria, and systematically collect maternal, placental, fetal, and neonatal data. Further research is also needed to clarify the optimal pregnancy-compatible antimicrobial regimen, the role and timing of mNGS in early diagnosis, the value of placental and fetal testing, and the long-term outcomes of infants exposed to maternal *C. psittaci* infection and antimicrobial therapy during pregnancy.

## Conclusion

To our knowledge, this is the first reported case of full-term delivery following gestational psittacosis. The favorable outcome emphasizes the importance of early diagnosis, pregnancy-compatible antimicrobial therapy, and multidisciplinary collaboration in the management of this severe infection. By providing a detailed clinical feature of clinical course and a systematic review, this report enhances the current understanding of gestational psittacosis and offers valuable insight for optimizing care in this high-risk population.

## Methods

### Case data collection and ethics

Clinical data for the present case were retrospectively collected from the patient’s medical records, including demographic information, exposure history, clinical manifestations, laboratory findings, imaging results, microbiological tests, treatment, maternal outcome, and neonatal outcome. The diagnosis of gestational psittacosis was based on compatible clinical features, a relevant avian exposure history, pulmonary imaging findings, and detection of *C. psittaci* by metagenomic next-generation sequencing (mNGS). This study was conducted in accordance with the Declaration of Helsinki and was approved by the Peking University Third Hospital Medical Science Research Ethics Committee. Written informed consent was obtained from the patient for publication.

### Literature search strategy

A systematic literature search was performed in PubMed and Embase from database inception to 22 March 2026. The search combined terms related to psittacosis and pregnancy. The PubMed search strategy was (“psittacosis” OR “*Chlamydia* psittaci” OR “Chlamydophila psittaci”) AND (“pregnancy” OR “pregnant” OR “gestational” OR “maternal” OR “placenta”). The Embase search strategy was adapted using corresponding Emtree terms and free-text keywords. Reference lists of relevant reviews and eligible articles were also manually screened to identify additional cases.

### Eligibility criteria and study selection

Studies were considered eligible if they reported individual patient-level data on gestational psittacosis or pregnancy-associated infection attributed to *C. psittaci* or related historical nomenclature. Eligible studies were required to provide extractable information regarding maternal clinical manifestations, gestational age, antimicrobial treatment, maternal outcomes, and/or fetal or neonatal outcomes. Case reports, case series, and literature reviews containing extractable individual cases were included. When duplicate cases were identified across multiple publications, only the most comprehensive report was retained for analysis.

Studies were excluded if they were unrelated to gestational psittacosis, included only non-pregnant patients, or reported exclusively animal or veterinary cases without human pregnancy data. Articles lacking sufficient individual clinical information for data extraction were also excluded, including conference abstracts with inadequate details and narrative reviews without patient-level data. In addition, studies for which full-text assessment or reliable data extraction was not possible were excluded from the final analysis.

### Article selection process

The database search identified 215 records, including 141 from PubMed and 74 from Embase. After removal of duplicates, 122 records were screened by title and abstract. Eighty-six records were excluded because they were conference abstracts, reviews or systematic reviews without extractable individual cases, unrelated research articles, non-relevant topics, or articles with inaccessible language or insufficient relevance. Thirty-six full-text articles were assessed for eligibility; four were excluded because key clinical features were unavailable and two were excluded for using other drugs. Overall, 30 cases of gestational psittacosis were included in the literature review.

### Data extraction and synthesis

For each eligible case, the following variables were extracted when available: maternal age, presumed exposure source, gestational age at first presentation, presenting symptoms, laboratory abnormalities, radiological findings, major complications, antimicrobial treatment, fetal outcome, maternal outcome, placental infection status, and evidence of fetal infection. Clinical manifestations and complications were defined according to the descriptions provided in the original reports.

### Statistical analysis

Categorical variables were summarized as counts and percentages, whereas continuous variables were presented as mean ± standard error of the mean (SEM), when appropriate. The gestational age at presentation was compared between cases with fetal survival and fetal death using an independent-samples t-test. All statistical analyses were two-sided, and a P value < 0.05 was considered statistically significant.

## Data Availability

The raw data supporting the conclusions of this article will be made available by the authors, without undue reservation.
